# easyExon – A Java-based GUI tool for processing and visualization of Affymetrix exon array data

**DOI:** 10.1186/1471-2105-9-432

**Published:** 2008-10-14

**Authors:** Ting-Yu Chang, Yin-Yi Li, Chih-Hung Jen, Tsun-Po Yang, Chi-Hung Lin, Ming-Ta Hsu, Hsei-Wei Wang

**Affiliations:** 1 Institute of Microbiology and Immunology, National Yang-Ming University, Taipei, Taiwan; 2 Institute of Biochemistry and Molecular Biology, National Yang-Ming University, Taipei, Taiwan; 3 Institute of Clinical Medicine, National Yang-Ming University, Taipei, Taiwan; 4 Veteran General Hospital-Yang Ming Genome Center, National Yang-Ming University, Taipei, Taiwan; 5 Department of Education and Research, Taipei City Hospital, Taipei, Taiwan

## Abstract

**Background:**

Alternative RNA splicing greatly increases proteome diversity and thereby contribute to species- or tissue-specific functions. The possibility to study alternative splicing (AS) events on a genomic scale using splicing-sensitive microarrays, including the Affymetrix GeneChip Exon 1.0 ST microarray (exon array), has appeared very recently. However, the application of this new technology is hindered by the lack of free and user-friendly software devoted to these novel platforms.

**Results:**

In this study we present a Java-based freeware, easyExon , to process, filtrate and visualize exon array data with an analysis pipeline. This tool implements the most commonly used probeset summarization methods as well as AS-orientated filtration algorithms, e.g. MIDAS and PAC, for the detection of alternative splicing events. We include a biological filtration function according to GO terms, and provide a module to visualize and interpret the selected exons and transcripts. Furthermore, easyExon can integrate with other related programs, such as Integrate Genome Browser (IGB) and Affymetrix Power Tools (APT), to make the whole analysis more comprehensive. We applied easyExon on a public accessible colon cancer dataset as an example to illustrate the analysis pipeline of this tool.

**Conclusion:**

EasyExon can efficiently process and analyze the Affymetrix exon array data. The simplicity, flexibility and brevity of easyExon make it a valuable tool for AS event identification in genomic research.

## Background

Alternative splicing of messenger RNA is a means of regulating gene expression and increasing proteome and functional diversity[1]. Identifying alternative spliced isoforms on a genomic scale was impeded by the lack of systematic tools such as splicing-sensitive microarray. Microarrays using exon-junction or exon-specific probes were used first for genome-wide alternative splicing analysis in yeast[2] and then to detect alternative splicing differences in mammalian tissues with a validity rate of about 50%[3] to 70–85%[4,5]. A genome-wide analysis of mouse transcripts using exon-orientated microarray and factor graphs revealed tens of thousands of potential new exons and reconciled discrepancies in current cDNA databases[6]. Genome-wide analysis of brain-specific splicing using microarray also revealed how a neuronal splicing factor, Nova, shapes the synapse[7,8].

Specific alterations in splicing patterns have been found in association with cancers, many of which may play functional roles in anaplasia, metastasis and invasion[9]. In the case of adenocarcinoma, alternative splicing forms of CD44 are expressed in the tumor part and overexpression of these splicing variants is associated with enhanced tumorgenecity[10,11]. In the case of astrocytoma, a switch of 2 alternative splicing forms of FGFR1 from a lower to a higher affinity receptor provide a growth advantage during turorgenesis[12].

Nevertheless, the mechanisms of splicing regulation and the exploration of different splicing forms in mammalian tissues are poorly understood in comparison with the well-explored signaling pathways that regulate transcription of functionally coherent sets of genes. Understanding the biologic significance of alternative splicing (AS) has been impeded by the difficulty in systematically identifying and validating transcript isoforms.

The AS events may sometimes be detected by classical gene-centric expression microarrays if the probes can discriminate the alternative spliced regions[13,14]. There is also a Bioconductor package, Splicegear, which can discover the AS event from the traditional expression microarray[15,16]. However, gene-centric expression microarrays are still not suitable for analysis the whole transcriptome since the analysis scale is constrained by the original probe design. Recently, the Affymetrix™ Inc. has developed a new exon-centric array that not only allows global analysis of gene expression but also includes detection and measurement of differential splice variation. This new type of microarray (GeneChip^® ^Exon 1.0 ST array; exon array in brief)[17] was designed to target all the annotated and predicted exons in human, mouse as well as rat genomes. Exon array for human contains more than 5.5 million features, corresponding to approximately 1.2 million exon clusters with over 1.4 million probesets. The probe select regions (PSR), the unit of non-overlapped exon, are defined by both existing annotation information and *de novo *prediction algorithms such as GENSCAN and TWINSCAN. As a result, exons can be virtually reassembled into over 250,000 transcripts according to a range of annotation sources.

New technology brings new knowledge, but it also increases the demand of new and robust analysis algorithms and bioinformatics tools for fast data processing and accurate interpretation. Currently, a few algorithms applicable to exon array technology, such as ANOSVA, GenASAP, MIDAS (Microarray Detection of Alternative Splicing), PAC (Pattern-Based Correlation), MADS (Microarray Analysis of Differential Splicing) or FIRMA (Finding Isoforms using Robust Multichip Analysis), have been developed for identification of global AS events[4,5,18-22]. Nevertheless, only few tools incorporating those algorithms are freely available, thus making the utilization of exon array difficult. Several free software, such as oneChannelGUI, exonmap/X:Map, have been developed for the analysis of exon array data. Both oneChannelGUI and exonmap are add-on Bioconductor packages designed to support fine-grained analysis of exon array data [23-25]. However, both packages require a certain degree of programming background, which is difficult for most biologists. Hence, a user-friendly tool is still required.

In this study we present easyExon, a stand-alone Java application, to provide a user-friendly platform for researchers who have no programming experience. EasyExon offers a standardized analysis pipeline for exon array, and is efficient in data handling. On the other hand, easyExon can also integrate with other related software such as the Affymetrix Integrated Genome Browser (IGB)[26] or the Partek™ GS Exon software package[27] for a more comprehensive analysis of exon array data. Human, mouse and rat exon arrays are all supported. This software combines probeset differences with exon information and provides users a comprehensive presentation of the AS events, thereby facilitating the subsequent wetlab experiments.

## Implementation

EasyExon is a Java GUI application that deploys Java Web Start technology to provide a flexible platform. It enables users to launch the most up-to-date version online and to analyze unpublished profiles locally. Owing to the mobility of Java language, the whole software can be launched in different platforms without restraint. This application has been tested in both Intel Pentium/Core 2 Duo/Xeon and AMD Athlon 64 CPUs on Windows XP/2003 32 bit version, Open SuSE Linux 10.3 32/64 bit versions and Mac OSX 10.5 of Intel architecture with Java 6 run-time environment installed. When analysis is conducted on the core probesets for a dataset of 10 arrays, which is a common scale in most experiments, each step described below takes only 5–10 seconds on a personal computer with Intel E6550 dual core CPU and 2G RAM running Windows XP professional 32 bit version. However, since some steps (*Steps 2–5*) require accessing to our annotation server, the processing time may depend on the internet connection. Users can improve the processing efficiency by installing a local copy of database and meta-probeset files. All the microarray data are processed on the user's own computer for the privacy protection. No information is shared.

If users want to acquire annotation information, easyExon can communicate with the MySQL annotation database server through MySQL Connector/J, a Java driver that converts Java Database Connectivity (JDBC) calls into the TCP/IP protocol for MySQL database connection. To speed up data processing, the probeset-to-exon information is pre-mapped and saved in the database server. We acquired the exon information from the refGene, ensGene and knownGene annotation tables from the UCSC Genome Bioinformatics[28]. Users can choose the gene annotation they are accustomed to for visualization. We applied the most up-to-date Hg18/Mm8/Rn4 versions for annotation. Results are visualized by JFreeChart, an open-sourced Java chart library, which displays the line charts of probeset intensity distribution, splicing index, fold change, as well as area chart of exon information (see below).

EasyExon can also employ other exon array-related software to expand its analysis power. For example, by automatically calling the Affymetrix Power Tools (APT)[29] at the background, easyExon can produce summary files when user uploaded .CEL files as input data. EasyExon also enables users to launch the Affymetrix Integrated Genome Browser (IGB) before graphic presentation. Moreover, each filtrated transcript can be displayed on IGB by clicking the hyperlink on the left panel of our application interface.

**Figure 1:**
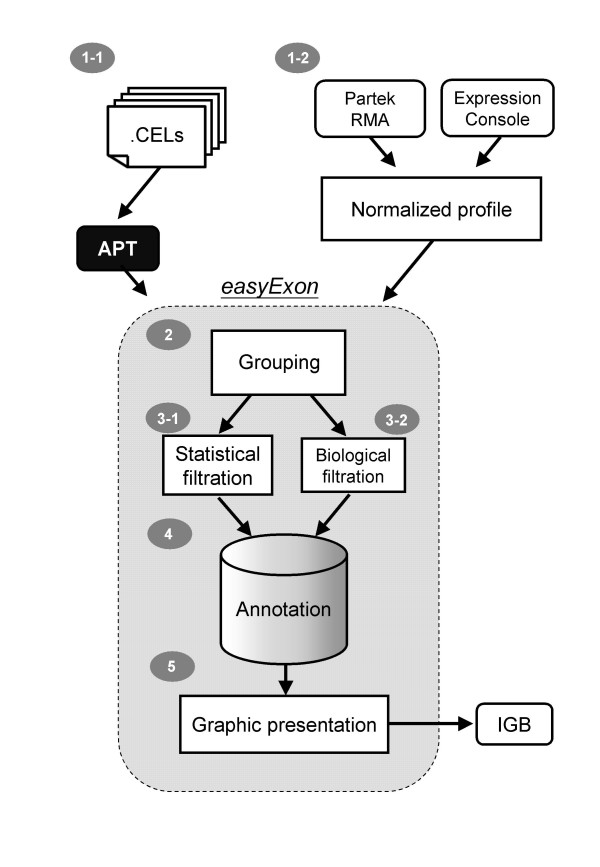
**Implementation of easyExon**. Analysis can start from .CEL files if users choose to download the APT executives when launching easyExon (*Step 1-1*). Users can also start from normalized profiles produced from Affymetrix Expression Console or Partek™ GS (*Step 1–2*). After array grouping (*Step 2*), the input array data are subjected into the feature filtration step (*Step 3-1 *and/or *3-2*). The filtered transcript clusters are annotated through the annotation database (*Step 4*). The final step (*Step 5*) comprises of gene visualizations and provides hyperlinks to IGB. The first 4 steps are similar to standard microarray analysis, while *Step 5 *is specific for exon array.

This software exhibits several unique presentations and user-friendly elements by following five simple steps (Fig. 1):

### Step 1: Data preparation

EasyExon can process the raw intensity files (*.CEL files) from the beginning (Fig. 1, *Step 1-1*). After data loading, gene/exon signal summarizations are calculated automatically by calling the Affymetrix Power Tools (APT) package, which can execute the binary file "apt-probeset-summarize" to generate both exon level and gene level summary files. Two of the most commonly used signal estimation algorithms, RMA[30] and PLIER[31], were implemented to combine information from probes belonging to the same transcript, or exon, to generate expression signal value of the gene or exon. Presence/absence of an exon is determined by the "Detection Above Background" (DABG) algorithm directly from .CEL files using surrogate background intensities. A small number (16 by default) is added to each summarized value to stabilize the variation on low intensity probesets before log_2 _transformation. The above protocol is according to the Affymetrix White Paper for exon array[32].

**Figure 2:**
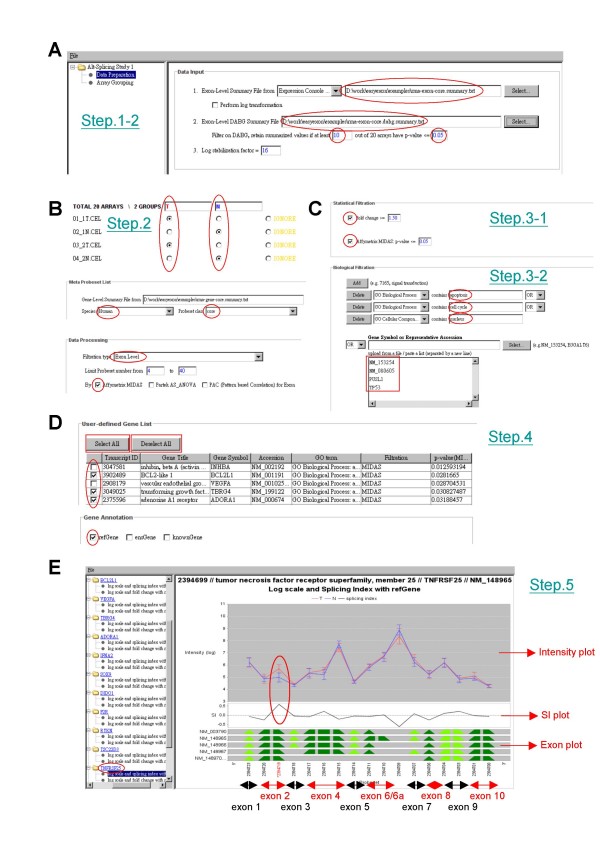
**An easyExon pipeline for analyzing exon array data**. (**A**) *Step 1-1*, the data input interface. Users have to specify the path of exon-level normalized profile, an optional DABG file (circled in red). (**B**) *Step 2*, array grouping. The gene-level summary file is loaded in this step. Users can also specify the array type, meta-probeset profile, exon/gene level of filtration and statistical methods. (**C**) *Step 3*, statistical and biological filtration. Exon and gene expression level can be filtered by fold change and/or p-value from the previous selected statistical methods. Users can also search for genes of interest by specifying GO categories or typing in gene IDs. (**D**) *Step 4*, annotation. This table implements with a "user-defining" interface for users to select or de-select features. (**E**) *Step 5*, graphic presentation of analysis results. This figure includes the probeset intensity plot (*upper*), SI (Splicing Index) plot (*middle*) and the exon annotation information (*lower*). In the exon annotation plot, exons are separated by different colors.

Another option is to import a normalized and summarized file produced by other tools such as the Affymetrix Expression Console package or other GeneChip^® ^compatible software (e.g., Partek™ GS program) (Fig. 1, *Step 1–2*, Fig. 2A). Both the normalized signal matrix file (exon level or gene level) and an optional exon level DABG matrix should be tab delimited text files with chip names in columns and probeset names in rows.

### Step 2: Array Grouping and Data processing

After uploading the required files, array samples is assigned into two groups in the "Array Grouping" panel (Fig. 1, *Step2*, Fig. 2B). Microarrays to be excluded in later analyses can be assigned to the "Ignore" groups.

The normalized matrix is organized and loaded for further analysis. EasyExon by default initiates a 1024 M memory heap size, which is suitable for the analysis with more than 100 exon arrays on one single launch of easyExon. The optional DABG p-value can serve as quality control for probeset signal. By default, if half of the total arrays show insignificance on one probeset, this probeset will be marked gray in the later graphic presentation.

The connection between "exon signal" and "gene signal" is determined by the meta-probeset file (Hg18/Mm8/Rn4 meta-probeset mappings) (Fig. 2B). Affymetrix offers three different classes of meta-probeset files according to different annotation strategies. Generally, "core" and "extended" classes are more convincing since they are deduced from experimental evidences. The "full" class contains mainly computational predicted regions and is therefore suitable for discovering new transcripts and exons. The meta-probeset information can be loaded from our web server directly through easyExon (Fig. 2B). Users can also include their own meta-probeset files into easyExon for further analysis.

### Step 3: Feature filtration

#### Statistical filtration

We developed Java classes to adopt the Affymetrix MIDAS and PAC alternative splicing algorithms for statistical filtration according to the Affymetrix White Paper[32]. The smallest MIDAS p-value for each exon in a transcript cluster regards as the representative p-value. The results from Partek AS ANOVA for exon level filtration are also supported. Users can use multiple selections to choose more than one method at the same time (Fig. 1, *Step 3-1*; Fig. 2C). Splicing index (SI)[2] and intensity fold change is also calculated and used later (see *Step 5*).

#### Biological filtration

Molecular biologists sometimes would like to know if there are any AS events associated with a specific biological function. In such cases, users may pre-filtrate AS events according to the corresponding Gene ontology (GO) annotation[33]. Users can enter either GO IDs or GO terms (exact or partial match) to monitor the AS event before or after statistical filtration (Fig. 1, *Step 3-2*; Fig. 2C). Moreover, users can enter or upload IDs of interested genes (Gene Symbol, Refseq or Genbank ID) to visualize their expression profiles at the exon level (the non-mapped identifiers are displayed to inform users) (Fig. 2C).

### Step 4 and 5: Annotation and Graphic Presentation

#### Gene annotation information

The transcript clusters that passed statistical and/or biological filtrations are presented in a brief annotation table according to the supporting data from Affymetrix (Fig. 1, *Step 4*; Fig. 2D). Note that each transcript cluster may contain more than one accession number but only the representative one and its corresponding gene symbol, as well as its filtration strategy, are shown. Users can manually select or de-select genes for graphic presentation (Fig. 2D). The mini or full annotation table can be exported from easyExon for further analysis. The corresponding probeset signal files can also be exported as *.egr or *.gff format to the Affymetrix IGB browser for viewing the transcript information.

#### Graphical view

The normalized, log-transformed and variance stabilized probeset intensities of a transcript is plotted as signal mean ± standard error in an intensity plot (Fig. 1, *Step 5*, Fig. 2E). Probesets marked with asterisks stand for statistically significant AS events which pass the user-defined MIDAS/Partek AS ANOVA threshold. Probesets marked with "PAC" indicate the correlation coefficient (r-value) is smaller than the defined PAC threshold. Probesets colored in red represent the intensity fold change between groups is greater than the threshold (default is 1.5 folds) (Fig. 2E). Probesets in grey indicate the confidence of probeset signals is poor according to the DABG filtering strategy (defined in *Step 1*).

**Figure 3:**
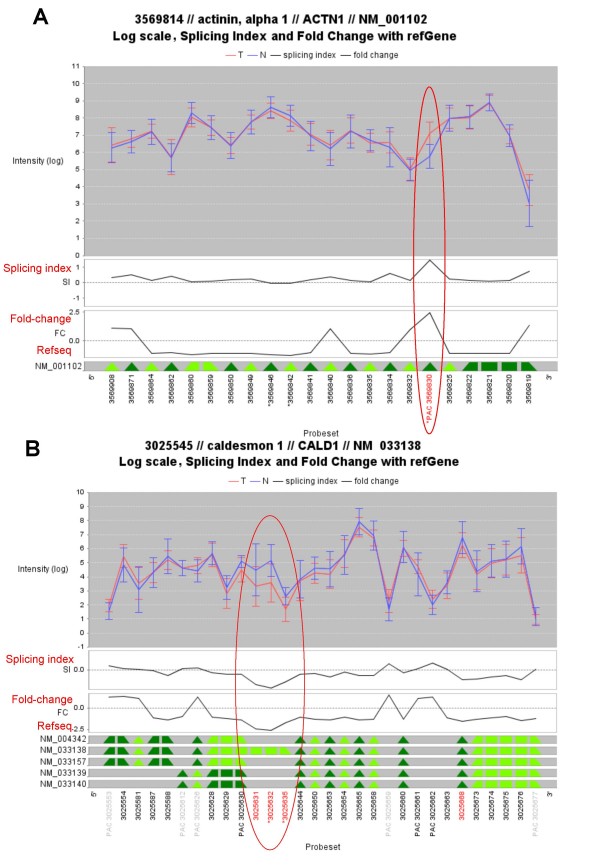
**Applying easyExon on a public colon cancer dataset**. (**A**) The transcript cluster of ACTN1. The 19^th ^exon is upregulated in the tumor part of colon cancer (the red line). This exon is targeted by probeset 3569830 which is significant in both MIDAS (labeled with an asterisk) and PAC (labeled with "PAC") tests. The fold change of this probeset between groups is greater than 1.5 (in red). (**B**) The transcript cluster of CALD1. The 3' end of the 6^th ^exon is downregulated in the tumor part. This region is targeted by probesets 3025631, 3025632 and 3025635 (circled in red) and is identified by MIDAS (labeled in red).

The degree of alternative splicing can also be visualized by the Splicing Index (SI) plot in the same panel. The side-by-side visualization of SI plots and intensity plots will facilitate the selection of AS events by users. If the intensity ratio of a probeset between 2 groups is different from the average ratio of the whole transcript cluster, the SI value will shift away from 0 (Fig. 2E, circled in red). Alternatively, the correlated fold change of a probeset is plotted across the whole transcript cluster for the reference of AS analysis (see Fig. 3).

#### Exon information

To correlate probeset intensity profile to the known transcript structure, we acquired genome coordinator from the refGene, ensGene and knownGene annotation tables. The mapping is based on Hg18/NCBI build 36 for human, Mm8/NCBI build 36 for mouse and Rn4/RGSC v3.4 for rat from UCSC Genome Bioinformatics[28]. The transcript structure of a given gene from these three depositories is plotted together with the intensity plot, SI or FC (fold change) plot at a 5' to 3' orientation (Fig. 2E). Probesets of the same exon are grouped together and represented by a triangle or trapezoid. Adjacent exons are demonstrated with different colors (Fig. 2E). Therefore, whether the AS event occurs within a known exon or outside the known exon regions can easily be observed. Users can identify whether a specific isoform is up- or down-regulated in one sample group by the intensity plot (Fig. 2E). In this example transcript (TNFRSF26, ID 2394699), the second probeset (ID 2394719, in red) targeting exon 2 is up-regulated in colon cancer samples (Fig. 2E). The expression fold change of this probeset is larger than 1.5 (labeled in red) and the difference is statistically significant (marked by an asterisk). This indicates there are two isoforms: one with both the 5' and 3' region of exon 2 and another with only the 5' region. The former isoform is upregulated in the tumor part.

## Results and Discussion

### Alternative splicing events in colorectal carcinoma

Here we illustrate an example of applying easyExon to quickly identify AS events by analyzing a public accessible colorectal carcinoma dataset, which includes 10 pairs of tumor and normal samples[34]. From that dataset 5 novel splicing events affect cytoskeletal organization (ACTN1, VCL, CALD1, CTTN, TPM1), 2 events affect extracellular matrix proteins (FN1, COL6A3) and a single event participates in integrin signaling (SLC3A2) were identified and confirmed by RT-PCR experiments[35]. All those novel isoforms are in our filtrated PSR list (fold change ≧ 1.5 folds, p-value ≦ 0.08), with ACTN1 and CALD1 being the most significant ones (p-value = 0.022 and 0.052, respectively). Actinin (ACTN1) is a component of stress fibers and links the cytoskeleton to adherent junctions. It may facilitate the detachment of focal adhesion and therefore enhance migration ability [36-38]. Caldesmon 1 (CALD1) can bind to actin and induce stress fibers and focal adhesions under the control of calmodulin[39]. The corresponding graphs of these AS events are shown in Fig. 3.

### Comparison with other exon array-driven tools

#### (1) exonmap/X:Map

The raw intensity files of exon array can also be analyzed and visualized through a Bioconductor[15,16] package, exonmap, and an associated genome browser, X:MAP[23,24]. The output information is quite detail and this application can demonstrate genome browser-liked plots of user-defined genes. Intensity information of probesets can also be labeled with different colors. However, the drawback is that when utilizing the exonmap package on the public accessible colon cancer dataset[34], it can only process part of the total dataset at the normalization step, due to memory limitation (even on a 64-bit computer running 64-bit versions of Linux and R.). Moreover, the annotation infrastructure suggested is somehow complicated and needs some programming background. Users also have to construct a database offered by the original group. Both tasks are difficult for most molecular biologists. EasyExon thereby provides a more user-friendly environment for the analysis and interpretation of Affymetrix exon array data. MIDAS p-value can be calculated by the "*splanova*" function in the exonmap package. But again users will have to manually execute several commands to obtain the analysis results. Our tool therefore becomes a much more handy choice for most biologists who have no or little programming experience.

#### (2) oneChannelGUI

Another GUI tool which can analyze exon array data is oneChannelGUI[25], it is also a package of Bioconductor. Although relatively easy, this package requires two additional Tck/Tk libraries, BWidget and TkTable, to be properly installed before analysis can be started. Again this will be an obstacle for most biologists. Also, the speed and memory consumption of R is a limitation for analyzing large scale data, such as those from exon array. In easyExon, we designed a specialized data structure to efficiently process exon array data, thereby save time and computing power for users. Although OneChannelGUI can handle the whole colon cancer dataset in the format of raw .CEL files or normalized tables, it only outputs an annotation list of potential alternative spliced transcripts. Further comprehensive demonstrations of AS events, such as graphic presentation of alternative spliced exons or implementation with genome browser, are still required. We incorporated such functions in easyExon for helping users to interpret their data more easily.

### The future of easyExon

It is always essential to verify drylab data by wetlab techniques. For exon array, RT-PCR confirmation of filtrated exons is a crucial step before conducting any further biological experiments. It is necessary to design cross-exon primers for the interested exons. Currently, exon sequences can be acquired through IGB, Ensembl or other genome databases but the whole process is time-consuming. To facilitate the primer design step, we will integrate sequence information into our graphic presentation window.

In addition to the human/mouse/rat Exon 1.0 ST arrays, Affymetrix has also released GeneChip^® ^Gene 1.0 ST arrays (gene arrays in brief) for these 3 species. Similar to exon array, gene arrays also allow researchers to interrogate the entire length of the gene, not just the 3' end[40]. On gene arrays each of the well-annotated genes is represented by approximately 26 probes spread across the full length of the gene. Thus, this new type of whole-transcript array also holds the potential for researchers to analyze expression profiles in an exon-wise manner. So far the default pipeline for gene array analysis is to automatically summarize all 26 probes into a single expression value per gene, allowing standard analysis software packages and simple analysis workflows to be used to analyze data. However, by re-organizing probe targeting regions to exon information available in Refseq, Ensembl or other repositories, it is possible to create an exon array-like analysis pipeline for gene array data in easyExon for measuring exon expression levels. This will allow users to acquire at least two different types of transcriptome information by performing only one array experiment.

## Conclusion

EasyExon provides a user-friendly, platform-independent and efficient processing way to explore Affymetrix exon array data. It offers a standardized pipeline for data analysis. By statistical filtration implemented with MIDAS and PAC, users can find a list of AS candidates; by biological filtration integrated with GO information, users can examine if any category of genes have AS events. EasyExon is specialized on the graphical presentation of user specified transcript clusters to demonstrate the differential expressed exons. Taken together, easyExon combines several filtration strategies and exon information and provides users a comprehensive presentation of AS events, thereby facilitating the following wetlab experimental design.

## Availability and requirements

Project name: easyExon

Project home page: 

Operating system: Platform independent

Programming language: Java

Other requirements: Java 1.60 or higher

License: free

## Abbreviations

AS: stands for alternative splicing; SI: stands for splicing index; and FC: stands for fold change.

## Authors' contributions

HW and TC conceived the data analysis pipeline. TC, TY and YL designed the user interface. TC, CJ, TY, CL, MH, and HW suggested desired features and algorithmic approaches. YL, TY and CJ carried out the implementation. The online documentation and manuscript were written by TC, YL and HW. All authors read and approved the final manuscript.
